# S.W. Orthopaedic Club

**Published:** 1991-06

**Authors:** 


### South West Orthopaedic Club

### Spring Meeting at Exeter April 20th 1991

The X-ray quiz was won by Mr Hossein Medhian. The South West Orthopaedic Club prize Certificate was awarded to Mr Kevin O'Dwyer for his paper "Tibial shaft fractures with an intact fibular'', and the certificate was presented on behalf of the Club by Professor Robin Ling.

The afternoon session consisted of the clinical papers, abstracts of which follow, and following the business meeting a guest lecture was given by Professor Robin Ling entitled "Cement — Friend or Foe''. The club dinner was held in the evening at the Forte Hotel in Exeter.

In the papers that follow the authors are in the correct order, but it is important to state that the Author who read the paper is the one who is marked by an asterisk.

### INVESTIGATION OF THE INTERNAL MECHANICS OF INTERVERTEBRAL DISCS USING STRESS PROFILOMETRY

**D.S. McNally,* M.A. Adams, A.E. Goodship,**
Comparative Orthopaedics Research Unit, University of Bristol

The gross mechanical properties of intervertebral discs have been thoroughly investigated, but comparatively little is known about the internal mechanical functioning of the disc tissues themselves. A technique was developed for measuring the stress within loaded cadaveric intervertebral discs.

A strain gauged membrane mounted on the side of a 1.3mm diameter needle was pulled through the disc at constant speed. The orientation of the membrane was changed by rotating the needle, so that profiles of vertical and horizontal components of compressive stress could be obtained. Study of these profiles allowed regions of the disc ("functional annulus'' and "functional nucleus) to be defined on the basis of their mechanical behaviour rather than their appearance. It was possible to measure the dimensions of these areas with an accuracy of ±2mm, and the magnitude of the stress to ±4%. There was no evidence that repeated measurement of stress profiles, (except in extremely degenerated discs), caused any damage or perturbation of the disc tissues.

Stress profiles varied considerably between discs and were highly dependant on the severity of degenerative changes. They showed that the mechanical behaviour of individual disc tissues was dependant not only upon their location, but also upon the loading and loading history of the disc.

By correlating the mechanical behaviour of disc tissues determined using stress profilometry with the structural and biochemical properties of the tissues a greater understanding of the mechanisms of disc function and failure may be achieved.

### A COMBINED ANTERIOR AND POSTERIOR RESECTION AND SPINAL STABILIZATION FOR ANEURYSMAL BONE CYST

**Hossein Mehdian* and Christopher Weatherley**
Princess Elizabeth Orthopaedic Hospital, Exeter

A 19 year old boy with a 6 month history of pain and stiffness in the upper cervical region was found to have a lytic lesion involving the anterior and posterior elements of C2, C3 and C4 causing a severe Gibbus deformity at C3.

Clinical examination revealed that neck motion was limited in all directions by pain and muscle spasm. Neurological examination was normal. Roentgenogram studies revealed a lytic expansile lesion involving the posterior elements of C2, C3 and C4 with a complete collapse of C3, partial involvement of C2 and C4 vertebral bodies, producing a severe Gibbus deformity. Bone scan revealed diffuse increased uptake at the level of the lesion and routine laboratory studies were negative.

The patient was admitted to hospital and a plan for two stage procedure was made.

The patient was placed in skull traction prior to surgery and through a posterior approach open biopsy of the lesion was performed and tumour was excised and spinal stabilization and fusion with instrumentation was carried out from C1–C5.

Two weeks later, while the patient was held in skull traction, through a right sided anterior approach, the remaining tumour was excised and the spine was stabilized with a tricortical iliac bone graft from C2 to C4.

The patient had an uncomplicated post-operative course and maintained on a Foster frame for a period of three weeks and then immobilized in a Minerva cast.

The final pathological diagnosis was aneurysmal bone cyst.

The patient remained pain free obtaining full correction of the deformity and has had no recurrence to date.

The purpose of this report is to emphasize that the involvement of several adjacent cervical vertebrae by an aneurysmal bone cyst is unusual and that a complete excision of the tumour with combined anterior and posterior procedures will minimize the rate of recurrence and neurological involvement.

### A REMOVABLE CIRCUMFERENTIAL ANKLE FOOT ORTHOSIS FOR CONTROLLED EARLY MOBILISATION FOLLOWING ACHILLES TENDON RUPTURE: A PROSPECTIVE RANDOMISED TRIAL:

**M. Saleh, P.D. Marshall,* R. Senior and A. MacFarlane**
From Cardiff Royal Infirmary and the Northern General Hospital, Sheffield

Early mobilisation may be beneficial in the repair of closed ruptures of the Achilles tendon. A circumferential orthosis was designed which controls the foot in 15° of equinus and is removable for controlled movement during physiotherapy.

To accommodate this position of moderate equinus, the orthosis is used in conjunction with an insole within an extra-depth shoe, which provides a heel raise of 2.5 cm. The orthosis is used following an initial period of three week's immobilisation in a below knee cast, and full weight bearing in the orthosis is permitted immediately.

A randomised prospective trial was conducted to compare twenty patients managed using the orthosis with a group of twenty patients managed in the established conservative manner of cast immobilisation for eight weeks. Patients managed using the orthosis regained mobility significantly more rapidly ($P<0.001$) and found it to be more comfortable than cast immobilisation. Assessment at three, six and twelve months post injury consistently revealed that the range of dorsiflexion at the ankle joint was significantly greater in the orthosis treated group ($P<0.001$), there being persistent restriction of dorsiflexion in the cast treated group. Despite the use of early active and passive mobilisation there was no evidence of overlengthening of the tendon. There was one re-rupture in each group.

### JOINT REPLACEMENT, DENTAL SURGERY & ANTIBIOTIC PROPHYLAXIS. A SURVEY OF CURRENT PRACTICE

**Andrew Grant, Ceri Hoddinott**
Dept. Orthopaedics and Trauma, Cardiff Royal Infirmary

Late infection about prosthetic joints remains a cause of great morbidity and indeed mortality. The meticulous care taken at the time of insertion of the joint arthroplasty can be undermined by subsequent haematogenous infection. Transient bacteraemia secondary to dental manipulation has been implicated as a cause of late infection following joint arthroplasty. Although remaining unproven, there are numerous case reports in the literature supporting the association. The administration of prophylactic antibiotics during dental manipulation would provide protection from this potential source of infection.

A survey of 125 each of local Orthopaedic surgeons, Dental surgeons and General Practitioners was undertaken to assess the practice of prescribing prophylactic antibiotics in patients with prosthetic joints undergoing dental manipulation. The results showed a high percentage of surgeons (10 per cent) with late sepsis in prosthetic joints attributed to dental disease or manipulation. Although 52 per cent of surgeons stated they routinely advised the use of prophylactic antibiotics during dental manipulation, only three per cent of General Practitioners conclude that the problem of sepsis of dental origin appears more common than expected and the need for prophylactic antibiotics in dental treatment must be more efficiently communicated to both patients and Dental surgeons.

### THE CONSERVATIVE MANAGEMENT OF TRANSVERSE FRACTURE OF THE SACRUM WITH NEUROLOGICAL FEATURES

**S. T. Phelan, Dai Anthony Jones, Michael Bishay**
Morriston Hospital, Swansea

Four cases of transverse fracture of the sacrum with neurological symptoms and signs are presented. These cases demonstrate the typical features of such fractures, and these are:
1) The diagnosis is delayed.
2) There are radiological difficulties in making a diagnosis.
3. The indications for surgery in such cases are not well defined.

The cases discussed, whilst having neurological complications, responded very well to conservative management and it is recommended that whenever fractured sacrum is suspected clinically that specific sacral X-rays are taken in order to confirm diagnosis, and that even when complications are present conservative management is recommended.

### TIBIAL SHAFT FRACTURES WITH AN INTACT FIBULA

**K. J. O'Dwyer, L. DeVriese, H. Feys, L. Vercruysse, D. C. Jameson-Evans**
Princess Elizabeth Orthopaedic Hospital, Exeter

A retrospective study of 281 conservatively treated adult tibial shaft fractures was undertaken. All patients were aged between 15 and 50 years. The association between high velocity and/or open fractures and delayed union was confirmed. Fractures with an intact fibula occurred in 16% of cases and were noted to have a lower incidence of delayed union than comparable fractures with a fractured fibula.

A further study of 68 tibial shaft fractures with intact fibulas was then undertaken. Various factors which may be associated with delayed union were studied including age, velocity of injury, associated wounds, site, fracture morphology, displacement and angulation. The only factors noted to delay union were 1) open fractures, 2) high velocity injuries, 3) oblique or comminuted fractures. Displacement and angulation played a less significant role but were still associated with delayed union. No other factors examined were associated with delayed union.

Of particular interest was the behaviour of the fracture during treatment. Fractures initially in neutral tended to remain in neutral. Those however that were initially in varus tended to revert to varus despite a good initial correction following manipulation.

The presence of an intact fibula has a beneficial effect on tibial shaft fractures. Conservative treatment is in general advocated although those initially in varus should be considered for surgical treatment because of the risk of both delayed and mal union.

### THE SURGICAL MANAGEMENT OF METASTATIC LESIONS OF THE SPINE

**J. L. Fowler, H. Mehdian, C. R. Weatherley**
Princess Elizabeth Orthopaedic Hospital, Exeter

This is a review of the last 20 consecutive cases of spinal metastases referred to the Spinal Unit. The present symptoms were severe pain or neurological deterioration or both. In several cases the initial presentation of the disease was the spinal deposit and delays in diagnosis and referral were common.

The aims of surgery were the relief of pain and the preservation or recovery of neurological function. In most cases there was significant pain relief and in no case did surgery lead to a neurological deterioration. Functional recovery was seen following surgery but not in those cases previously treated by radiotherapy.

There is a case for the surgical management of metastatic spinal tumours and early diagnosis and referral is important. Collaboration of the surgeon and the radiotherapist and oncologist is important as clearly is the timing of any adjuvant therapy.

